# Race, ethnicity, and considerations for data collection and analysis in research studies

**DOI:** 10.1017/cts.2024.632

**Published:** 2024-10-29

**Authors:** Sima Sharghi, Shokoufeh Khalatbari, Amy Laird, Jodi Lapidus, Felicity T. Enders, Jareen Meinzen-Derr, Amanda L. Tapia, Jody D. Ciolino

**Affiliations:** 1 Department of Biostatistics and Computational Biology, University of Rochester Medical Center, Rochester, NY, USA; 2 The Michigan Institute for Clinical and Health Research, University of Michigan, Ann Arbor, MI, USA; 3 Oregon Clinical and Translational Research Institute, Oregon Health and Science University, Portland, OR, USA; 4 Department of Quantitative Health Sciences, Mayo Clinic, Rochester, MN, USA; 5 Department of Pediatrics, Cincinnati Children’s Hospital Medical Center, University of Cincinnati College of Medicine, Cincinnati, OH, USA; 6 Department of Quantitative Health Sciences, Division of Clinical Trials and Biostatistics, Mayo Clinic, Rochester, MN, USA; 7 Department of Preventive Medicine – Biostatistics, Northwestern University Feinberg School of Medicine, Chicago, IL, USA

**Keywords:** Race, ethnicity, data collection, analysis, generalizability

## Abstract

Research studies involving human subjects require collection of and reporting on demographic data related to race and ethnicity. However, existing practices lack standardized guidelines, leading to misrepresentation and biased inferences and conclusions for underrepresented populations in research studies. For instance, sometimes there is a misconception that self-reported racial or ethnic identity may be treated as a biological variable with underlying genetic implications, overlooking its role as a social construct reflecting lived experiences of specific populations. In this manuscript, we use the We All Count data equity framework, which organizes data projects across seven stages: Funding, Motivation, Project Design, Data Collection, Analysis, Reporting, and Communication. Focusing on data collection and analysis, we use examples – both real and hypothetical – to review common practice and provide critiques and alternative recommendations. Through these examples and recommendations, we hope to provide the reader with some ideas and a starting point as they consider embedding a lens of justice, equity, diversity, and inclusivity from research conception to dissemination of findings.

## Introduction

Research studies involving human subjects typically carry a requirement to collect and report on demographic data pertaining to race and ethnicity. However, there is a lot of variability and minimal guidance on best practices for doing so. Common practices can propagate issues and misunderstanding around race and ethnicity. Race and ethnicity are not reliable indicators of genetic differences and their use in genetic research and medical practice can lead to inaccuracies and reinforce social biases.

The conduct of a study, from conception of the research question to dissemination of findings, involves many decisions that should be considered through a lens of justice, equity, and inclusivity. Data equity [1] frameworks offer principles and practices to approach decisions through this lens. In this manuscript, we consider the We All Count (WAC) data equity framework [2] (see supplemental file for a list of abbreviated words), which organizes a data project into seven stages: Funding, Motivation, Project Design, Data Collection and Sourcing, Analysis, Reporting, and Communication and Distribution. We focus on data collection and analysis, and we provide examples of typical practice, illustrate why they may or may not be optimal, and provide considerations and recommendations for alternative practical approaches. We unpack issues with current practices in collecting, analyzing, and reporting race and ethnicity data through many of our own experiences and lessons learned in conducting research studies.

We assert that self-reported race and ethnicity should not be used as a marker or surrogate for genetics, as they represent a social construct rather than a biological one, reflecting lived experiences [3,4]. There is a need to collect and report on race and ethnicity in research studies, but researchers should use these variables in analyses purposefully with caution. In the sections to follow, we (1) set the context for collection and analysis through considerations of research goals and audiences, (2) provide examples of and critique data collection instruments for collecting race and ethnicity, (3) unpack meanings and inferences when using race and ethnicity variables in analyses, (4) touch on reporting and downstream implications, and conclude with some general recommendations for researchers to consider.

## Background and considerations

The study objectives and specific hypotheses should determine data collection and analysis plans. Starting from study inception, the role of race and ethnicity should be considered carefully. Specific questions the researcher may want to ask themselves as they plan their studies include: (1) Does race or ethnicity play a direct role in the study objectives? (2) How might we define race and ethnicity in the population of interest? (3) What is the hypothesized distribution of race and ethnicity in the study population of interest? (4) What is the best way to ascertain these data from the population of interest? (5) How should these variables be used in analyses to address the research question(s)? The response to these questions will help shape data collection and analysis, and as the manuscript progresses, we aim to provide the researcher with options as a starting point for collection and analyses as they consider these questions.

### Research question considerations

Beginning with the research question, if the answer to question (1) above points to a direct role for race and ethnicity in the research to accomplish study objectives, the research question should reflect this role: whether it is characterizing an association where race or ethnicity may be a potential confounder or an effect modifier, revealing health disparities or sources of structural racism, or something else. For instance, a study titled “Racial Inequities in Access to Ventricular Assist Device (VAD) and Transplant Persist After Consideration for Preferences for Care” by Cascino, T. M *et al*. [5] goes beyond descriptive summaries, and it intends to explore a hypothesized systematic bias within heart failure (HF) patients. Aside from some relevant demographic and clinical data, patients’ preferences and desire for receiving advanced therapies were also recorded to examine bias among clinicians toward patients who identify as Black. As a result of careful hypothesis generation, collection of appropriate data, and sound analyses, the authors conclude: “Among patients receiving care by advanced HF cardiologists at VAD centers, there is less utilization of VAD and transplant for Black patients even after adjusting for HF severity, quality of life, and social determinants of health, despite similar care preferences. This residual inequity may be a consequence of structural racism and discrimination or provider bias impacting decision-making.”

In contrast, other studies may not have an aim of evaluating disparities using race or ethnicity directly. For example, “Efficacy and Safety of a Quadruple Ultra-low-dose Treatment for Hypertension (QUARTET USA)” sought to evaluate efficacy of a therapy in a hypertensive population [6–8]. Although race and ethnicity are not central to the study’s main aims, collecting and analyzing these demographic factors is important for providing contextual information in dissemination.

### Audience considerations

Aside from considering the goal and the hypothesis of the research collecting these data, it is important to consider the audience for dissemination of the work. The audience may include the funders, regulatory bodies, researchers, clinicians, participants, or the public. Consider participant perspectives first. Transparency in data use, analysis, and reporting is key to building trust and collaboration. There are excellent resources on equitable and inclusive data practices that can be helpful for deciding how to frame research and research findings appropriate for a given audience [9,10].

#### Funder

As stated in WAC framework [2], in the funding stage we need to map out the relationship between data, resources, and authority within the project: “The power structures involved in a data project need to be revealed, evaluated and sometimes altered in order to get the equity that everyone – not just stakeholders or data workers, but funders too – is looking for.” Thus, in the WAC framework, the design, conduct, analysis, and reporting of a project must cater to and make sense for all those involved, including the funder. We begin this discussion with the funder as one “audience” that the researcher must consider.

We use the National Institutes of Health (NIH) as one example of a common funder among US-based human subjects research studies. The NIH requires both (a) an inclusion of minoritized individuals and underserved population statement (with justification for any exclusions as applicable) and (b) an enrollment table that summarizes enrollment projections overall and racial and ethnic categories. Thus, the principal investigator must collect and report on race and ethnicity, according to the categories set forth by the funder. Refer to Table 1 for an example “Table Shell.” At minimum, regardless of other plans for reporting and goals of the research, the investigator conducting NIH-sponsored research would need to collect these data.

**Table 1.** Example table shell for National Institutes of Health-funded human subjects’ studies

Cumulative (Actual)	Ethnic Categories									
	Not Hispanic or Latino			Hispanic or Latino			Unknown/ Not Reported Ethnicity			Total
Racial Categories	Female	Male	Unknown / Not Reported	Female	Male	Unknown / Not Reported	Female	Male	Unknown / Not Reported	
American Indian/ Alaska Native	0	0	0	0	0	0	0	0	0	0
Asian	0	0	0	0	0	0	0	0	0	0
Native Hawaiian or Other Pacific Islanders	0	0	0	0	0	0	0	0	0	0
Black or African American	0	0	0	0	0	0	0	0	0	0
White	0	0	0	0	0	0	0	0	0	0
More than One Race	0	0	0	0	0	0	0	0	0	0
Unknown or Not Reported	0	0	0	0	0	0	0	0	0	0
Total	0	0	0	0	0	0	0	0	0	0

One concern with this table is that the “Asian” category is broad. According to the Census Bureau, an Asian includes “a person having origins in any of the original peoples of the Far East, Southeast Asia, or the Indian subcontinent including, for example, Cambodia, China, India, Japan, Korea, Malaysia, Pakistan, the Philippine Islands, Thailand, and Vietnam.” We note that countries like Iran, Iraq, Afghanistan, and Armenia do not fit that definition and thus it is difficult to understand where study participants with origins from these countries may identify among these categories required by the funder [11]. We note that there are recent updates forthcoming to the US census and future federal forms whereby “Middle Eastern/North African (MENA)” has been added and further ethnicity and race have been combined into a single set of data elements, which we view as a positive step toward accurate representation of research study participants’ identities [12].

Based on a Pew Research report [13], “Six origin groups – Chinese (24%), Indian (21%), Filipino (19%), Vietnamese (10%), Korean (9%) and Japanese (7%) – accounted for 85% of all Asian Americans as of 2019.” This report depicts the rest of the countries that account for “Asian” categories in research studies in the US and they include 2% or less of the following: Pakistani, Thai, Cambodian, Hmong, Laotian, Taiwanese, Bangladeshi, Nepalese, Burmese, Indonesian, Sri Lankan, Malaysian, Mongolian, Bhutanese, and Okinawan. Thus, even among the Asian categories minoritized individuals exist, adding to the diversity of the lived experiences among those of Asian descent. Unfortunately, by default, this concern is overlooked in most research studies since the tendency is often to use the NIH-specific categories as outlined in Table 1.

The funder’s requirements for racial and ethnic data should align with research goals. NIH guidance mandates de-identification but doesn’t specify collection methods, whether self-reported or from sources like Electronic Health Record (HER). ^14^While this practice of reporting on racial minority groups in clinical trials has been commonplace since 1993, (“In 1993, the United States (US) Congress passed the National Institutes of Health Revitalization Act as part of an effort to improve enrollment of minority groups in clinical trials.” [15]) the challenges of doing so persist to this day.

#### Clinical research community

When we consider describing a participant cohort or understanding generalizability of the study findings, we often think of the typical “Table 1” of a research article[16]. In fact, standard reporting guidelines for randomized clinical trials [17] (RCT) and observational studies [18] stress the importance of providing tabulated clinical and demographic characteristics for the study participants. The general reasoning is to help the general research community – those who would need to use and interpret the studies – determine both generalizability and gaps. From the table of the participants, we can start to understand the general characteristics of the study population. Participant race and ethnicity are important variables in this regard, since the lack of diversity may result in limitations on generalizability of the study findings [16].

**Figure 1.**
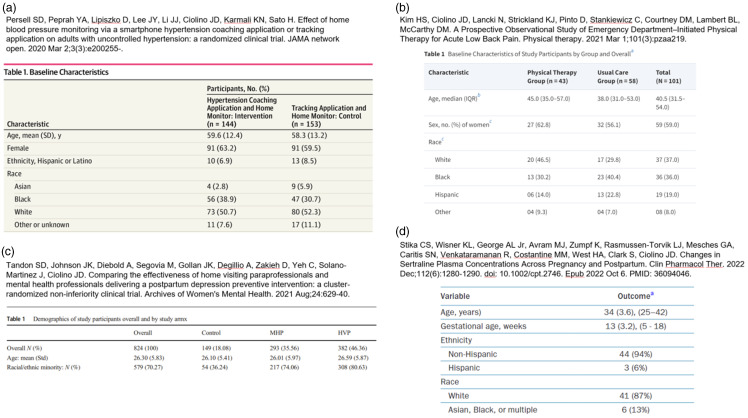
Examples of “Table 1” from several publications.

Figure 1 provides examples of several tables from one of the author’s collaborative portfolios [19–23]. We use these examples to illustrate the study-to-study variability in reports of racial and ethnic data. There is no single or perfect way to present these data, and each of the tables presented here carries its own pros and cons. Multiple considerations and discussions were involved in the development of these tables, and they underwent multiple iterations between coauthors, collaborators, reviewers, and editors prior to their eventual publication in the form illustrated here.

Here we outline some observations in typical practices (Figure 1).

- Panel (a) summarizes data from participants in a clinical trial on hypertension. As defined by the U.S. Census Bureau [24], race is categorized as American Indian or Alaska Native, Asian, Black or African American, Native Hawaiian or Other Pacific Islander, or White. However, in this table, the breakdown between unknown and other is not clear. Even though this categorization might be necessary for reporting purposes, the audience could benefit from knowing the exact race of the study participants.[25] For example, the authors could include in the text or a footnote, “those listed as “other” included XXX participants self-reporting YYY, etc.”
- Panel (b) summarizes data from study participants for a postpartum depression preventive intervention. It is difficult to understand the demographic profile of the study participants from the table alone. Race and ethnicity could be broken down further, as it is argued that individuals of certain races tend to receive less mental health interventions [25]. Additionally, the definition of “minority” or the identities included in the “minority” category should be outlined in the manuscript or table footnote.
- Panel (c) summarizes data from study participants of a prospective study, comparing physical therapy and usual care for the patients who have visited an emergency care unit. Again, race and ethnicity categories could be expanded or fully described to help the audience understand the participant population more clearly. We do note here that race and ethnicity are not separated into different variables.
- Panel (d) illustrates participant characteristics in a cohort study evaluating changes in sertraline plasma concentrations across pregnancy and postpartum. Even though there are only six people in the category of “Asian, Black, or multiple,” it may be beneficial to certain audiences [26] to better understand this breakdown. In this case, the race variable was used for descriptive purposes only (i.e., to describe the study population), and due to the low cell counts and potential lack of anonymity that may be introduced in reporting these low cell counts in a table like this, the study team decided to report the self-reported racial identities in this way so that the reader would know that there were some participants identifying with these specific categories.

Practices in summarizing race and ethnicity data can exacerbate the marginalization of certain populations. For example, summarizations may be overly simplified (e.g., complex racial and ethnic identities can be grouped into broad categories that do not account for the unique lived experiences of smaller or mixed-race identities) or marginalized groups may develop a feeling of invisibility if they are lumped into larger categories. Additionally, the “White” or “Non-Hispanic White” category is frequently listed first and used as the category to which other categories are compared, reinforcing damaging notions about belonging. In fact, we see this occur in some examples in Figure 1. In line with other guidelines and recommendations, we suggest alphabetical ordering for *both* data collection and reporting [27]. Refer to other reporting guidelines and discussions in Section 5.1. Considering the many audiences of research studies and appropriate reporting for those audiences is often helpful in determining how to set up data collection tools.

## Data collection

Collecting race and ethnicity data from study participants may carry potential pitfalls, such as generating feelings of confusion, exclusion, and marginalization. Participants may wonder why the information is needed and how it will be used, which can diminish their interest and investment in the study. The WAC [2] Data Equity Framework provides important considerations for data collection and sourcing to mitigate these issues.

**Figure 2.**
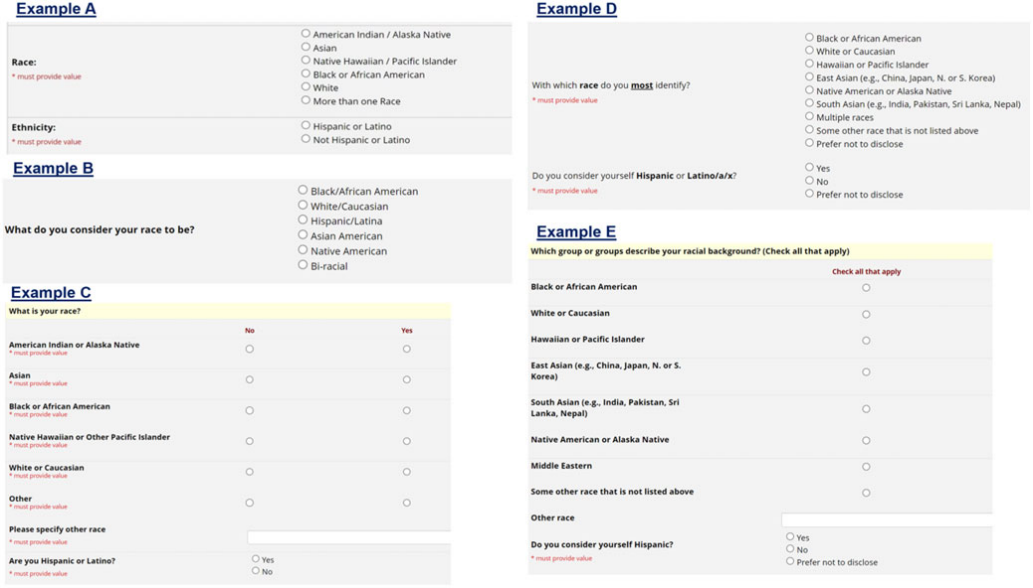
Examples of data collection form segments to solicit race and ethnicity information from a study participant.

From a study participant’s perspective, providing information on race and ethnicity can be problematic. The participant is the world’s expert on their identity. The framing of a question regarding these constructs can force the participant to misrepresent their own identity. We use Figure 2 to illustrate example data collection tools that may be used to solicit this information from a participant via a series of questions [28,29]. There are benefits and drawbacks to each of the options presented. For instance, Example A ensures consistent and concise data collection that would likely be required at minimum for a funder (Refer to Table 1); however, it requires the participant to select exactly one of the available options for race and ethnicity. A participant who does not identify with the race options listed must select from the available options, and someone who identifies with more than one option can register only if they identify with more than one option without the opportunity to list which ones.

Other concerns of the participants might be: “What is the difference between race and ethnicity? They seem to be asking the same thing from my perspective.” Or, “These categories feel very general. How can you group people of Indian, Chinese, Japanese, Korean, Thai, Indonesian (and many other) descent into just one broad category?” Possible considerations to address these concerns may be (1) Consider whether it is necessary to break the two constructs of race and ethnicity apart; refer to the aforementioned updates at the federal level on this idea [12] – researchers could consider using a set of fields as in Example B; (2) Ask a question that does not specifically state “ethnicity” in the label…“Do you consider yourself Hispanic?” (as in Example D or E); (3) Consider collecting data on a more granular level noting that it may be possible to collapse into larger, “required” categories later. This is preferred over risking participant difficulty with answering questions.

Identifying race and ethnicity can be particularly problematic for people who identify as American Indian/Alaska Native. Since most people who identify as American Indian/Alaska Native also identify with another racial group, American Indian/Alaska Native identity is often not registered when information on race is collected, resulting in a severe undercount of American Indian/Alaska Native people [30]. Moreover, many who identify as American Indian/Alaska Native also identify as Hispanic; a recent movement to treat Hispanic identity as a racial group rather than an ethnic one would mean that some who identify as American Indian/Alaska Native would be separated from that identity in research studies [30]. The way the US government currently handles race and ethnicity data separates more than three-quarters of people who identify as American Indian/Alaska Native from this identity. Such erasure of this identity exacerbates marginalization of these populations in research.

While pitfalls in collecting race and ethnicity data abound, they can be avoided. In a recent publication from results of an RCT, “Effect of Ivermectin vs Placebo on Time to Sustained Recovery in Outpatients with Mild to Moderate COVID-19: a Randomized Clinical Trial” [31] the authors explained the reason for its collection: participants were asked about race and ethnicity “due to the disparity in the burden of COVID-19 infection carried by marginalized communities based on race and ethnicity.” They also stated how these data were collected: “Participants were asked about ethnicity separately from race and were able to select any combination of race designations, including the option to not report any designation.” The demographic summary of the study population gave an authentic reflection of the race and ethnicity data participants provided. It separated ethnicity and race, reported each race designation as an independent binary variable, and presented race designations alphabetically rather than by number who marked each designation. The authors presented each race designation in the table without collapsing designations and used the opportunity to acknowledge the lack of representation in these designations as a limitation of the study: “while the inclusion criteria allow for a broad study population, this study failed to achieve the level of representation desired for underrepresented populations in terms of racial and ethnic diversity.”

Since collecting racial and ethnic information on study participants is necessary to understand inclusivity and generalizability of research, it is important for study personnel to collect this information accurately and to report it faithfully. Here we reiterate some existing recommendations [32].

1. *Clearly state rationale for collecting race and ethnicity information.* Understanding why race and ethnicity information is needed for the study and how it will be used conveys respect for the participant and builds trust. This rationale could look like: “Collecting information on race and ethnicity helps us understand our study participants’ background. It will help people reading the study results understand whether the study may or may not apply to them or their population of interest.”
2. *If providing a list of options:*
3. *Allow participants to select all that apply.* Participants who identify with more than one racial identity should be given the opportunity to enumerate these.
4. *Allow participants to not respond.* When participants are allowed to select “choose not to answer” for these questions or to skip the question(s) altogether, a sense of autonomy is instilled in the participants.
5. *Consider including an “other [specify]” option.* Participants who do not identify with any of the listed options should have the opportunity to register a different identity. Note we recommend specification of “other” even though this may provide inconsistencies with data entry and open up opportunities for error but may be worth the effort.
6. *Consider including an “unsure” option.* Participants who are unsure of what the question is asking or how to respond should be given the opportunity to register their uncertainty.
7. *Plan to acknowledge limitations to any approaches/results interpretations in any pieces of dissemination*.

When participants understand the importance of providing demographic information and can represent their identity accurately, they are more likely to participate. Careful consideration of racial and ethnic data collection in study design ensures participants feel seen and accurately represented.

In summary, race and ethnicity should always be collected in human research studies to allow for assessment generalizability. The reason for “why” and “how” these data are collected should be clear and communicated to the participants. Further, collecting and monitoring these data allows us to uncover potential systemic biases in enrollment to ensure the researchers do not unknowingly exclude participants of a certain background.

## Analysis

As aforementioned, it is important to consider the audience and the goal of the research in mind in every stage of a research, from inception to dissemination. With respect to race and ethnicity, researchers should ask themselves “What is the ultimate message we want to convey with respect to these data?” [33] “Are we adjusting or controlling for potential confounding effects, and if so, why might confounding by self-identified race or ethnicity be present in our study?” “Are we concerned about heterogeneity of effects, in particular health disparities by self-identified race and/or ethnicity?” These questions are especially important as researchers look to choose the right statistical analysis strategies. Consider the following examples.

### Hypothetical example – controlling for ethnicity versus heterogeneity of effect

To illustrate the statistical meaning behind “controlling for race or ethnicity” we use a hypothetical example. Consider a research study in which study participants are randomized into one of two interventional arms: Arm 1 = cognitive behavioral therapy (CBT); Arm 2 = treatment with antidepressant. In the hypothetical example, participants are followed for 2 years and have study visits every 6 months. The primary outcome of interest is depressive symptom severity score, and the primary aim is to evaluate intervention effects on depressive symptom scores over time. The overarching question is whether treatment with CBT is more/less efficacious than treatment with antidepressants in reducing depressive symptom scores over time. The general frequentist hypothesis framework for statistical analyses would involve the following hypotheses: H0: mean depression score for patients treated with CBT = mean depression score for patients treated with antidepressants, versus H1: mean scores in the two patient groups are not equal.

To compare depressive symptom scores over time, one may use a longitudinal model akin to the following: Y = intercept + time + study arm + error. For simplicity, we do not present all the details of the statistical analyses, variance components, and assumptions on this model, but we use a simplified and heuristic model to outline concepts. The primary statistical hypothesis test focuses on the regression coefficient for the “study arm” variable, treated as an indicator or a factor in the analytic model.

There are times, however, when researchers may need to “control” or adjust for racial or ethnic variables in this model. This would likely be a secondary or exploratory analysis, or the purpose may be to increase precision in estimating the main effect. To determine whether adjustment is merited, one should always go back to the research hypothesis and determine if it makes scientific sense to perform additional analyses controlling for race or ethnicity. In this example, assume we have reason to believe self-identified ethnicity does influence outcome (in this case depression scores), and it is scientifically justified to consider adjusting for ethnicity.

**Figure 3.**
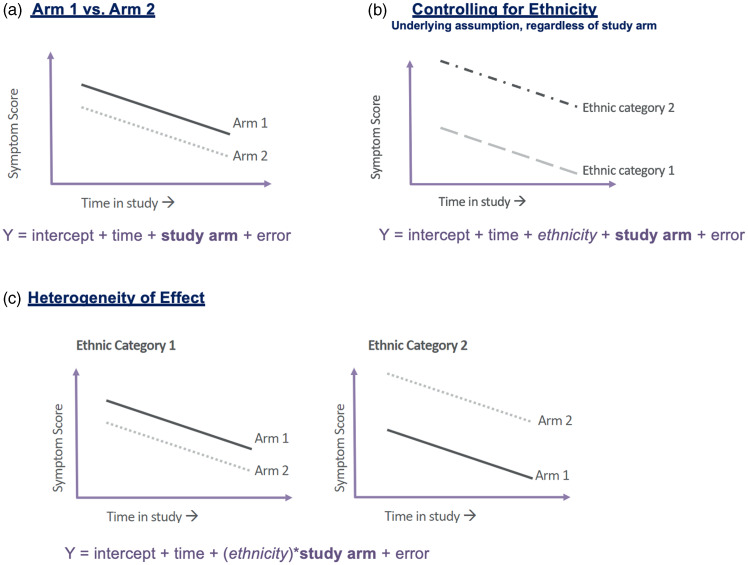
Visual of hypothetical example involving controlling for ethnicity versus heterogeneity of treatment effect.

Refer to Figure 3 for a visual depiction of the overarching hypothesis to be tested (A) and for a visual of the underlying assumption when “controlling” for ethnicity (B). We note that in this example, we are excluding the baseline measurement period from the visual, as in theory, the mean outcome value would be the same at baseline across both study arms. In this scenario lies an assumption of an inherent difference in depressive symptom scores for patients of different self-identified ethnic backgrounds, regardless of study arm. The hypothetical model becomes Y = intercept + time + *ethnicity* + study arm + error, where ethnicity would be treated as a factor or categorical variable within the model. The primary interest in this scenario (B) is still the study arm coefficient estimate and the associated hypothesis test. However, the inclusion of the ethnicity variable would only increase precision of this estimate if the assumption in Figure 3b holds.

Now if one were to desire to evaluate whether the intervention has varying effects for different ethnic identities (i.e., examine heterogeneity effect), we might respecify the model to something similar to the following (Figure 3c): Y = intercept + time + (ethnicity)*study arm + error. The inclusion of the interaction term between ethnicity and study arm seeks to evaluate evidence of a differing study arm effect within groups of participants self-identifying as specific ethnic categories. In our hypothetical illustration (Figure 3c), those identifying with ethnic category #1 tend to respond better with intervention from arm #2, while those identifying with ethnic category #2 tend to respond better with intervention from arm #1.

Taken together, when considering racial or ethnic variables in analyses we recommend the reader (1) revisit their research strategy and the overall study goals, hypotheses, and design; (2) review the role that self-identified race and ethnicity would play in the analyses and inference and determine the underlying scientific premise and statistical hypotheses of interest, (3) review the distribution of the categorical variable in question prior to conducting the analyses. We illustrate the reasoning for #3 with another example to follow. The reader should keep in mind that even if analyses are a part of the study goals, they may not be possible as group counts may be too low.

### Another example (based on a real study) – controlling for race

This example is based on a real study, but details have been removed or modified slightly to retain anonymity. Assume we have a study like the one in Section 4.1, and we would like to control for race in analyses. We pre-specify this plan to adjust for self-identified race in analysis. However, of 88 participants enrolled in our study, four participants self-identify as Asian; one as Black; five as multiple races; and 78 (89%) self-identify as White. Statistically, the largest subgroup provides the most “information” in the model. Larger subgroups tend to result in increased precision and more stable model estimates. If we leave these categories as is and put “race” as a four-category factor into our statistical model, we will most likely end up with nonsensical parameter estimates and confidence intervals (model instability issues) due to low cell counts. Anonymity may also suffer in reporting (refer to Figure 1d for an example of this scenario). Now suppose we decide to collapse these categories to the following: Asian/Black/multiple races: 10 (11%) and White: 78 (89%). If we include a two-level categorical variable in our analytic model, the model convergence or stability issues subside; however, referring back to the underlying assumption in the model (for example, see Figure 3b), adjusting this new variable implies that there is reason to believe that those identifying in the broad category of either Asian, Black, or multiple races would have inherently different depressive symptom scores than those identifying as White. It also implies that those falling into one of those broad groups have depressive symptom score behavior that would be more like one another than they would be to those of the larger subgroup. Referring back to the recommendation on considering adjustment in analyses, the underlying scientific premise of such an analysis would not be justified, and we should not adjust for race in this example. Ultimately, while it may be acceptable practice to “collapse” categories for descriptive purposes (especially due to anonymity issues and perhaps with some caveats), it would very seldom be appropriate to collapse racial categories for analyses.

Similarly, Naggie *et al*.[31] found that structural racism and resource access likely affect COVID-19 symptom duration. Despite broad inclusion criteria, their study on ivermectin lacked racial and ethnic diversity, revealing barriers that prevent minority participation.

### Using race and ethnicity in observational studies

In observational studies, race and ethnicity are often crucial for adjusting potential confounders, which are more prone to bias than in randomized controlled trials (RCTs). These variables often correlate with social and economic factors, potentially confounding key exposure-outcome relationships.

For example, if we were to use observational data to evaluate use of CBT versus treatment with antidepressants for depression using observational data rather than in the RCT from Section 4.1, we would likely need to control for several socioeconomic variables that might be related to both exposure (CBT vs. antidepressants) and outcome (depressive symptom scores). Some of these variables such as health literacy, income, education level, access to care, insurance status, etc. may not be measurable within a dataset or there may be inherently missing data on these variables. While there would not necessarily be an underlying scientific premise that race or ethnicity itself would be one such confounder, this variable may be related to these other important confounders.

It is common practice in observational studies to estimate a propensity score (PS) for exposure and control for that PS in primary analyses evaluating exposure and outcome relationships. PS estimation relies heavily on the types of covariates included in the model. While earlier suggestions advocated including all measured covariates in PS modeling [34], new empirical simulation-based studies recommend a more selective approach. For instance, Brookhart *et al*. (2006) [35] argue that only variables related to both exposure and outcome should be included since this increases precision without adding bias. Recent methodologies advocate for careful consideration of which covariates are included – race and ethnicity being prime examples. When deciding whether to include self-identified race or ethnicity as covariates in a PS model, investigators must consider what these variables represent within their analysis. Are they serving as proxies for other influential factors such as insurance status, area deprivation index, or income? Or do they capture additional unmeasured effects essential for understanding exposure-outcome relationships? If self-identified race or ethnicity has direct relevance to the outcome under study, their inclusion can enhance model accuracy; however, if their relevance is indirect – acting through other variables like socioeconomic status – they may not need inclusion unless residual confounding is suspected. Thus, include race and ethnicity in PS models based on their specific role. If they act as proxies for other factors, include those instead. If they capture unique aspects of the exposure-outcome relationship, their inclusion is justified for a thorough analysis.

### General considerations for analysis

We emphasize that these hypothetical examples focus on the social construct of self-reported identity, not biological or genetic relevance. They illustrate key considerations for researchers using these variables in analyses.

Aside from the recommendations provided above ([1] revisiting the goals, [2] understanding the scientific rationale, and [3] considering whether inclusion of race or ethnicity is viable in analyses), we list additional recommendations – many of which can already be located in an existing recommendations document – when conducting analyses outlined below [33].

First, when working with race or ethnicity variables in statistical models, small categories can lead to computational challenges, imprecise estimates, and model instability. Privacy concerns may arise when reporting or analyzing these small categories. In such cases, collapsing categories can help maintain anonymity and facilitate analyses. However, it is essential to align categories with the overall goals and consider contingency plans for analyses or reporting.

Additionally, the aforementioned document [33] includes several suggestions and options for researchers to consider with respect to parameterization of race and ethnicity variables in analytic models (assuming there is justification for including them based on overarching goals). They include (1) ensuring that racial and ethnic variables are included as a factor variable (i.e., not an ordinal or continuous variable), and (2) considering use of indicator variables (a series of yes/no or 1/0 variables depending on how the data were collected) for inclusion in models.

When choosing a reference category for categorical variables, avoid defaulting to the largest group, often “White” and “Non-Hispanic” in US studies. Consider using the first or last alphabetical category instead. Be mindful of the inferential implications when analyzing racial and ethnic variables.

## Other considerations and suggestions

### Reporting

Throughout the present manuscript, we focus on unbiased and equitable data collection and sound analyses. However, it is important to acknowledge the importance of carrying these ideas through to the report as the end result of this data collection and analysis is eventual report and dissemination of research findings.

Summarizing race and ethnicity data can be difficult when the data collection process is unclear. Often, summaries don’t explain how data were gathered, which can lead to biases, especially if the data are sourced rather than self-reported. This lack of clarity can distort results, reflecting the choices and biases of the data handlers, rather than accurately representing the study population.

We refer the reader to the (The Journal of the American Medical Association) JAMA guidance on reporting race and ethnicity and to additional guidance on using inclusive language in reporting [27,36]. We highlight some specifics here noting that the overarching goal of the JAMA guidance is to “…provide recommendations and suggestions that encourage fairness, equity, consistency, and clarity in use and reporting of race and ethnicity in medical and science journals [27].”

We list some general ideas presented in the JAMA guidance in Table 2.

**Table 2.** Recommendations from JAMA guidance on reporting [27]

Recommendation from JAMA Guidance	Comments in the context of this manuscript
Reporting of race and ethnicity should not be considered in isolation; It should be accompanied by other sociodemographic factors, social determinants; Intersectionality of race and ethnicity with other factors should be included.	Remember to refer to the overarching aims of the research. If race and ethnicity variables are not important for the overall research goals, one may consider omitting them or using other variables instead. If race and ethnicity variables are of importance, then consider using other factors in addition to or in place of these variables as the guidance suggests.
Inclusion of race and ethnicity in reports of medical research remain important for addressing concerns about racism, health disparities and inequalities.	The need to collect and report on race and ethnicity, at minimum, is often required. Using them in analyses may not always be sound, and researchers should again refer to our recommendation of returning to the aims, understanding the inference, and potentially adapting plans as needed.
Language and terminology must be accurate, clear, and precise. Language must reflect fairness, equity, and consistency in use and reporting of race and ethnicity.[36] Specific categories are preferred over collective terms, when possible; report the specific categories used and recognize that these categories will differ based on the databases or surveys used, the requirements of funders, and the geographic location of data collection or study participants. Categories included in groups labeled as “other” should be defined. Categories should be listed in alphabetical order in text and tables. Avoid collective reference to racial and ethnic minority groups as “non-white.” If comparing racial and ethnic groups, indicate the specific groups compared.	The same principles should be applied in data collection and analysis. Conceptualizing the eventual report and dissemination with these recommendations in mind may help with planning data collection and analysis. In other words, starting with the ultimate end product as a goal and working backward may help ensure consistency from the beginning through to the end of the study.
Report should include who identified participant race and ethnicity and the source of the classifications used (e.g., self-report or selection, investigator observed, database, electronic health record, survey instrument). If race and ethnicity categories were collected for a study, the reasons that these were assessed also should be explained in the Methods section. If collection of data on race and ethnicity was required by the funding agency, that should be noted.	In general, we recommend using participant self-report for race and ethnicity and other sociodemographic or social determinant variables. All details around justification for and methods of collection and analyses for race and ethnicity data should be pre-specified and eventually reported in the methods section of any final report. If there are space limitations preventing details, then supplemental materials (e.g., eMethods, protocol(s), manuals of procedures, analysis plans, etc.) should include these details and the primary report should refer to them.

### Impact of practices when handling race and ethnicity data

While the present manuscript focuses on issues around race and ethnicity in research, the eventual downstream effects of clinical research findings are changes in clinical practice. These can range from using predictive algorithms to understand patients’ risks and prognoses through optimal treatment strategies and precision medicine based on evidence from such studies. Recent literature [37] has illustrated the potentially devastating effects of algorithmic bias in medicine and race-adjusted tools, from triaging illnesses to quality of care received, and ultimately health outcomes for patients belonging to minority populations.

Examples of race-adjusted tools used are ubiquitous, across a multitude of fields and subspecialties, including risk calculators in cardiology, estimated Glomerular Filtration Rate as a measure of kidney function, Vaginal Birth After Cesarean risk calculation, urology risk calculators, and cancer survival prediction rates [38,39]. These risk calculators or scores consider race as a covariate to control for potential biological differences across self-reported race subgroups. Using race-adjusted scores has caused the over-consideration of race in referrals, treatment plans, and evaluations [20]. To reduce these downstream effects, race-adjusted tools should be avoided as they use race as a biological construct rather than a social construct, yet we know that race is a very poor surrogate for genetics/biology[3,4].

Shifting the paradigm away from race-based medicine and algorithms starts with shifting the paradigm in research. Considering and implementing the recommendations we put forth for precision and consistency in data collection, analysis, and reporting in research serve as one step in this process.

## Summary and discussion: what can researchers do?

This manuscript aims to start a dialog on collecting and analyzing race and ethnicity data in human research, offering initial options for researchers. It advocates for reducing bias in clinical and research practices and highlights the importance of recruiting diverse participants, despite challenges like cultural barriers and distrust, especially among non-US citizens.

We theorize that by asking themselves the following questions and thinking about them from the outset, researchers will be in a better position to continue to enforce a paradigm shift toward more equitable, diverse, and inclusive research when considering collection and analysis of race and ethnicity data:

1. Why are we collecting these data? Are they necessary to achieve the study objectives?
2. Does the way we are collecting these data allow us to accomplish the study aims?
3. Are there other sets of variables that would better address these aims, or are there additional variables to supplement race and ethnicity that would provide a clearer picture?
4. Do inferences in analyses make sense in the context of the study aims?

In all cases, researchers should have contingency plans and be adaptive (e.g., when analyses are no longer feasible based on the distribution of data) and be transparent. The daunting endeavor of reducing structural racism in medicine and in research cannot be solved with one study or one single group of investigators or clinicians, but it is a collective effort to move against an embedded set of processes. We hope that this manuscript and recommendations provided here help to continue the forward progress of recent work in this arena.

## Supporting information

Sharghi et al. supplementary material Sharghi et al. supplementary material

## Supplementary material

The supplementary material for this article can be found at https://doi.org/10.1017/cts.2024.632.
