# Supplementary material for: Race, ethnicity, and considerations for data collection and analysis in research studies
Source: J Clin Transl Sci. 2024 Oct 29;8(1):e182. doi: 10.1017/cts.2024.632 (PMC11626588; doi:10.1017/cts.2024.632)
Supplement: Sharghi et al. supplementary material [file S2059866124006320sup001.docx]

Table of the abbreviated words:

| **Complete Word** | **Abbreviated Word** |
| --- | --- |
| We All Count | WAC |
| Ventricular Assist Device | VAD |
| Heart Failure | HF |
| National Institutes of Health | NIH |
| Middle Eastern/North African | MENA |
| Electronic Health Record | EHR |
| United States | US |
| Randomized Clinical Trials | RCT |
| Cognitive Behavioral Therapy | CBT |
| Propensity Score | PS |
| estimated Glomerular Filtration Rate | eGFR |
| Vaginal Birth After Cesarean | VBAC |
| Justice, Equity, Diversity, and Inclusivity | JEDI |
| Biostatistics, Epidemiology, and Research Design | BERD |
| Special Interest Group | SIG |
| Association for Clinical and Translational Science | ACTS |
| The Journal of the American Medical Association | JAMA |
